# Long‐Term Stress Adaptation as a Highly‐Conserved Key Factor in Yeast Aging

**DOI:** 10.1111/acel.70513

**Published:** 2026-04-24

**Authors:** Yanzhuo Kong, Damola Adejoro, Christopher Winefield, Stephen L. W. On, Philip A. Wescombe, Arvind Subbaraj, Andrew Saunders, Venkata Chelikani

**Affiliations:** ^1^ College of Food and Chemical Engineering Shaoyang University Shaoyang China; ^2^ Faculty of Agriculture and Life Sciences Lincoln University Lincoln New Zealand; ^3^ Yili Innovation Centre Oceania Lincoln University Lincoln New Zealand; ^4^ National Center of Technology Innovation for Dairy Hohhot Inner Mongolia China; ^5^ New Zealand Institute for Bioeconomy Science Lincoln New Zealand; ^6^ Westland Milk Products Rolleston Canterbury New Zealand; ^7^ School of Science, STEM College RMIT University Melbourne Victoria Australia

**Keywords:** cell aging and yeast biology, cell survival, stress adaptation, stress response

## Abstract

Aging is commonly viewed as a passive consequence of accumulated damage; however, emerging evidence suggests that it may also represent an adaptive response to environmental stress. Here, we combined transcriptomic and metabolomic profiling of *Saccharomyces cerevisiae* to investigate how short‐term, long‐term, and recovery phases of stress exposure shape cellular physiology and lifespan. Short‐term stress‐induced protective pathways and longevity‐associated metabolites, including trehalose and 5′‐methylthioadenosine, consistent with enhanced stress resilience and proteostasis. In contrast, prolonged stress activated heat shock proteins and epigenetic regulators, coupled with metabolic signatures associated with loss of proteostasis, reduced energy homeostasis, and shortened chronological lifespan. Upon recovery, beneficial metabolites such as S‐adenosylhomocysteine were restored, highlighting the reversibility of stress‐induced aging trajectories. Phylogenetic analysis demonstrated conservation of these stress‐ and aging‐related genes across eukaryotes and prokaryotes, suggesting an evolutionary basis for aging as a long‐term stress adaptation. Together, these findings suggest that aging‐associated molecular changes are closely linked to conserved stress response pathways, with implications for understanding the hallmarks of aging.

## Graphical abstract

This study shows that long‐term, but not short‐term, stress exposure triggers molecular changes in yeast that mirror established hallmarks of aging, including altered proteostasis, epigenetic shifts, and reduced lifespan. These changes are reversible upon stress removal, and the genes involved are conserved across all domains of life.

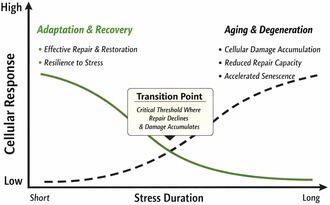

## 1 Introduction

What constitutes the fundamental and most basic aspect of the aging process (Poganik and Gladyshev 2023)? The formerly comforting notion that aging was a natural process involving older individuals gracefully making way for the younger generation for the benefit of the species was debunked by evolutionary biologists in the latter part of the twentieth century. They demonstrated that natural selection typically prioritizes individual reproduction over the well‐being of the species, rendering the idea that aging evolved to limit older individuals from reproducing for the benefit of the younger generation theoretically unsound (Williams 2001; Partridge and Gems 2002). Instead, it is now widely accepted that aging likely emerged later in biological history, and its precise origins and timing are central questions in evolutionary biology. Recent discoveries, such as aging in bacteria, suggest that aging predates the appearance of eukaryotic organisms, tracing its roots back to basic single‐celled life forms (Ackermann et al. 2003, 2007). No genes are known to have evolved specifically to cause damage and aging (Partridge and Gems 2002). Is there another potential evolutionary benefit to aging? Although natural selection on its own may not convincingly account for the development of aging, as it often leads to the demise of most individuals, it is clear that genes play a substantial role in the processes of aging and longevity. It is probable that the genes associated with aging and longevity are part of stress response systems (Lithgow and Kirkwood 1996). Nutrient and stress receptors play a role in prolonging lifespans triggered by a variety of environmental and physiological cues (Kenyon 2010). In‐depth research has highlighted that encountering short bouts of stress can enhance the way cells respond to stress, a phenomenon referred to as “hormetic stress.” This kind of stress contributes to extended lifespans by boosting the activities of molecular chaperones and other defense mechanisms. On the contrary, enduring periods of stress can overwhelm the body's compensatory responses, resulting in what is known as “toxic stress,” ultimately decreasing lifespan (Epel and Lithgow 2014; Yegorov et al. 2020). In mammals, dietary restriction and intermittent fasting paradigms have similarly demonstrated that stress‐adaptive metabolic responses profoundly influence health span and lifespan (De Cabo and Mattson 2019). According to the hyperfunction theory, and in the context of the updated hallmarks of aging framework (Lopez‐Otin et al. 2023), as cells age, they undergo notable transformations and deficiencies at multiple levels, indicative of their adaptation to a range of stressors (Eigenfeld et al. 2021; Blagosklonny 2012). A recent study by Poganik et al. reveals that biological aging could undergo temporary acceleration during periods of stress. However, this acceleration is temporary, and after the stress subsides, the biological age reverts to baseline (Poganik et al. 2023). This prompts the question of whether aging might potentially function as an adaptation mechanism to prolonged stress, rather than being solely a consequence of extended stress exposure. In this context, we hypothesize that the molecular and phenotypic changes observed during long‐term stress adaptation substantially overlap with established hallmarks of aging, and that these changes are at least partially reversible once prolonged stress is removed. We note that *Saccharomyces cerevisiae* also exhibits a finite replicative lifespan under ostensibly ideal conditions, representing a complementary aging paradigm with distinct pathways such as Sir2‐dependent asymmetric segregation of damaged proteins (Erjavec and Nyström 2007). Our hypothesis does not claim that stress adaptation is the sole mechanism underlying all forms of aging but rather that it constitutes a significant and conserved contributor. Gaining insights into the impacts of both short‐term and long‐term stress, as well as the recovery of cells from stress is essential to determining how much of the aging phenotype is contingent on sustained stress exposure. These findings may inform strategies to address or mitigate age‐related decline.

The budding yeast, *S. cerevisiae* , has served as a fundamental model organism for investigating cellular aging (Hotz et al. 2022; Nyström and Liu 2014). In particular, we believe yeast is an excellent model system to study the difference between short‐term stress and long‐term stress. Historically, studies focused on yeast aging were restricted to a narrow selection of genes and relied on hypothesis‐driven approaches that, while informative, may have constrained the scope of genes and pathways examined (Kaeberlein et al. 1999; Winzeler et al. 1999). These methodologies primarily involved the evaluation of specific genes based on existing knowledge or assumptions, alongside the exploration of additional traits such as stress resistance or alterations in gene expression associated with age‐related changes, which could potentially be linked to longevity (Kaeberlein et al. 2007). While single‐gene investigations in model organisms have previously advanced our understanding of aging (Kenyon et al. 1993), comprehensive genome‐wide inquiries into aging processes are now recognized as essential (Hughes et al. 2000; McCormick et al. 2015). This shift is driven by the recognition that a thorough understanding of aging requires a holistic approach, as genes are intricately interconnected, and multiple homologous genes arising from the ancient whole‐genome duplication may coexist in core metabolic processes like aging (Postma et al. 2022). The functional redundancy among paralogues, including redundancy between stress‐responsive transcription factor pairs such as *msn2/msn4* (Wu et al. 2021), can mask aging‐relevant phenotypes in single‐gene studies, necessitating the genome‐wide approach adopted in this current study. The chronological lifespan (CLS) of yeast is determined by assessing the duration for which cells remain viable during the late post‐diauxic and stationary phases after exponential growth. Research on yeast CLS, which serves as a model for understanding postmitotic cellular aging in more complex organisms, has been instrumental in discovering shared pathways and mechanisms regulating lifespan (Longo et al. 2012; Schroeder et al. 2013).

In this study, we utilized benzoic acid, a widely used food preservative and antifungal agent, at sublethal threshold levels to establish three distinct metabolic groups. The first group involved treating yeast cells with 10 mM benzoic acid for 24 h (Short‐term/ST Stressed Cells). The second group encompassed treating yeast cells with 10 mM benzoic acid for 500 h, with subculturing every 24 h (Long‐term/LT Stressed Cells). The third group consisted of LT Stressed Cells subsequently grown for 16 h without any benzoic acid, allowing for recovery (Recovered Cells). In this study, we used shorter chronological lifespan and reduced cell viability as phenotypic indicators consistent with accelerated aging, while recognizing that these measures alone do not constitute a comprehensive definition of the aging process.

We performed transcriptomic and metabolomic investigations, specifically emphasizing genes and metabolites that exhibited significant expression changes in cells subjected to long‐term stress (LT stressed cells). These included epigenetic regulators and secondary stress response genes, which are also recognized as key markers of the aging process (López‐Otín et al. 2013; Pasyukova et al. 2021; Zhang, Qu, et al. 2020). Additionally, we conducted a phylogenetic analysis of these genes to trace their evolutionary origins.

Our findings reveal notable differences in the transcriptomic and metabolomic profiles between LT Stressed Cells and ST Stressed Cells. The chronological lifespan of LT Stressed Cells was shorter than that of ST Stressed Cells, with several age‐related biomarkers being prominently expressed in the former. Several genes were specifically expressed in LT Stressed Cells, suggesting the existence of a potentially distinct mechanism, separate from the primary stress response, contributing to aging in these cells. Furthermore, our phylogenetic analysis of genes specifically expressed in LT Stressed Cells indicates high conservation, primarily from bacterial origins. This aging process observed in LT Stressed Cells seems to operate independently yet in harmony with stress response pathways, potentially offering valuable support for cell survival. Recovered Cells exhibited similarities to Control Cells, with several lifespan‐extension genes and metabolites activated in line with existing literature. Additionally, both Stressed and Recovered Cells produced several beneficial metabolites, hinting at potential biotechnological applications stemming from these cell states.

## 2 Results and Discussion

### 2.1 Short‐Term Stress Induces Protective Metabolites and Extends Lifespan

The body's longevity response to dietary restrictions and stress has been shown to be actively governed by pathways that sense stress and nutrients (Haq et al. 2022; Reiling and Sabatini 2006; Kenyon 2010). In model organisms, restricting methionine has been shown to extend lifespan and delay the onset of age‐related diseases. Previously, Ogawa et al. (2016, 2022) observed that the metabolite S‐adenosyl‐L‐homocysteine (SAH) activates adenosine monophosphate (AMP)‐activated protein kinase (AMPK) in yeast, leading to extended lifespan. Their recent research demonstrates that SAH supplementation can decrease methionine levels and reproduce many of the physiological and molecular effects associated with methionine restriction in *Caenorhabditis elegans* . Treating with SAH prolongs lifespan by activating AMPK and providing similar benefits to methionine restriction (Ogawa et al. 2016, 2022).

To further explore the impact of short‐term and long‐term stress on yeast, we utilized benzoic acid, a widely used food preservative and antifungal agent, at sublethal threshold levels to establish three distinct metabolic groups. At physiological pH, benzoic acid diffuses across the plasma membrane in its protonated lipophilic form and dissociates in the more alkaline cytoplasm, causing intracellular acidification. The cell responds by activating the plasma membrane H^+^‐ATPase (Pma1) to expel protons, imposing a significant ATP cost (Piper et al. 2001; Warth 1991). Additionally, the accumulation of benzoate anions disrupts membrane integrity and inhibits metabolic enzymes, creating a dual stress on energy homeostasis and membrane function. The first group involved treating yeast cells with 10 mM benzoic acid for 24 h (Short‐term/ST Stressed Cells). The second group encompassed treating yeast cells with 10 mM benzoic acid for 500 h, with subculturing every 24 h (Long‐term/LT Stressed Cells). The third group consisted of LT Stressed Cells subsequently grown for 16 h without any benzoic acid, allowing for recovery (Recovered Cells). RNA transcriptomics and metabolomics were then used to identify changes between the three groups in gene expression and metabolite production respectively.

**FIGURE 1:**
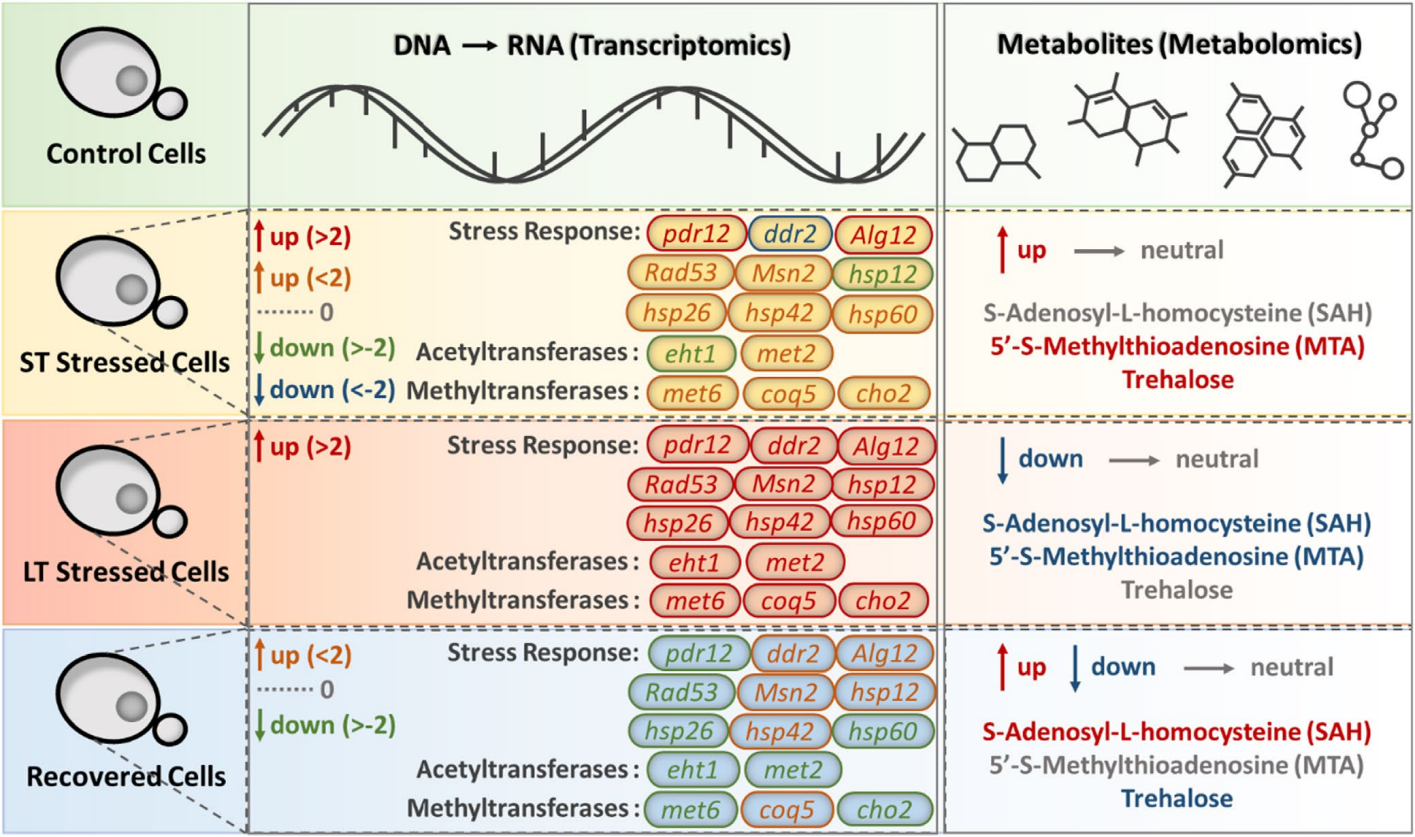
Summary figure. Transcriptomic and metabolomic changes in *Saccharomyces cerevisiae* key genes and metabolites in response to epigenetic modification. The labels for up‐ (red and orange) and downregulation (green and blue) in the transcriptomic section are determined using log_2_ fold change values obtained from the results of differential gene expression analysis. Control Cells: wild‐type *S. cerevisiae* ; ST Stressed Cells: short‐term stressed cells, *S. cerevisiae* exposed to 10 mM benzoic acid for 24 h; LT Stressed Cells: long‐term stressed cells, *S. cerevisiae* exposed to 10 mM benzoic acid for 500 h (24 h/subculture); Recovered Cells: *S. cerevisiae* exposed to 10 mM benzoic acid for 500 h (24 h/subculture), followed by growing in regular YPD broth for 16 h.

**FIGURE 2:**
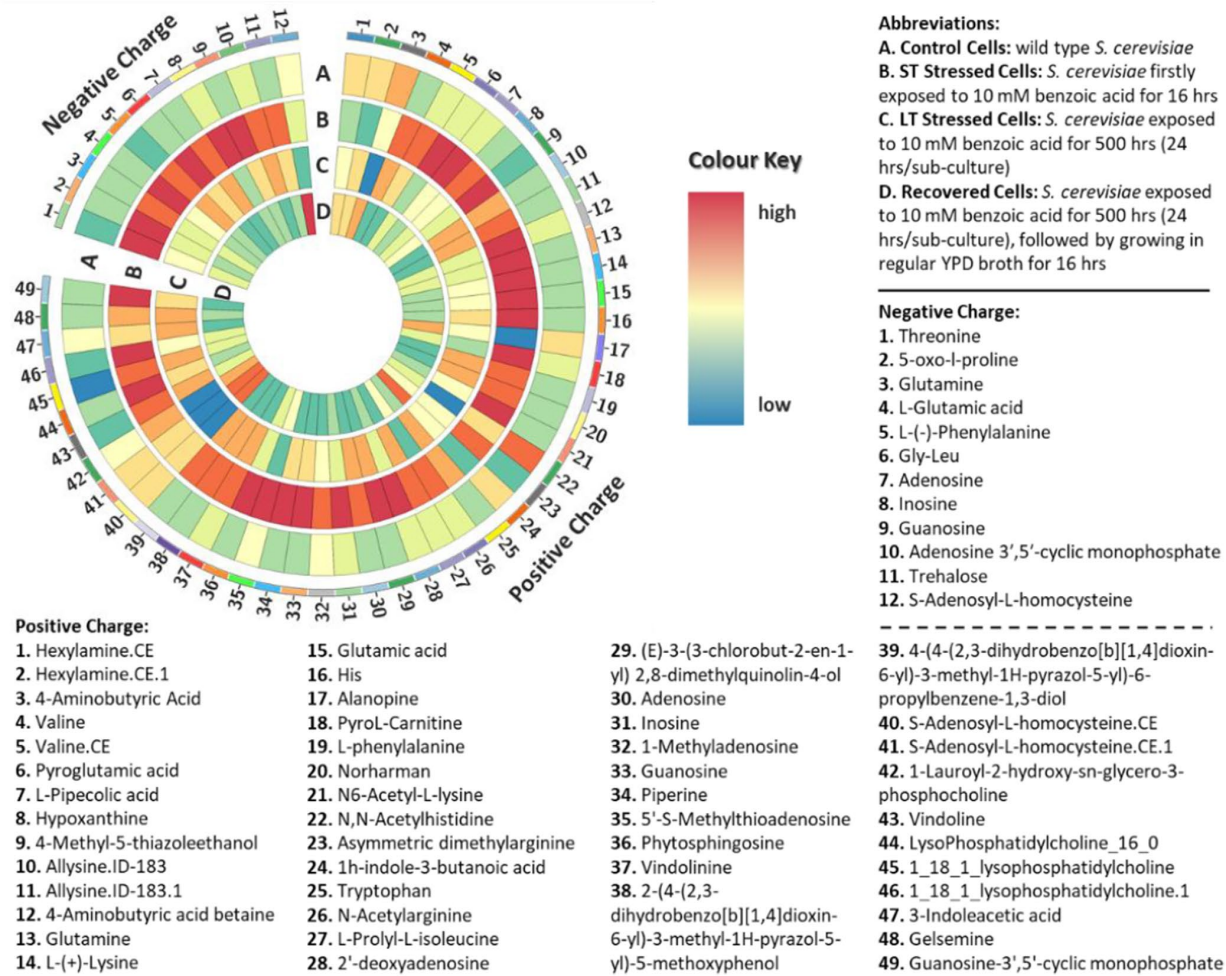
Circos plot of known metabolite concentrations detected by LC–MS/MS metabolomic analysis in control and treated *Saccharomyces cerevisiae* cells, including both positive and negative ion modes. The color scale reflects the relative abundance of each metabolite, represented as log_2_ fold‐change values across the different *S. cerevisiae* cell types. Red indicates the highest abundance (log_2_ fold change = 1.5), while blue indicates the lowest abundance (log_2_ fold change = −1.5).

**FIGURE 3:**
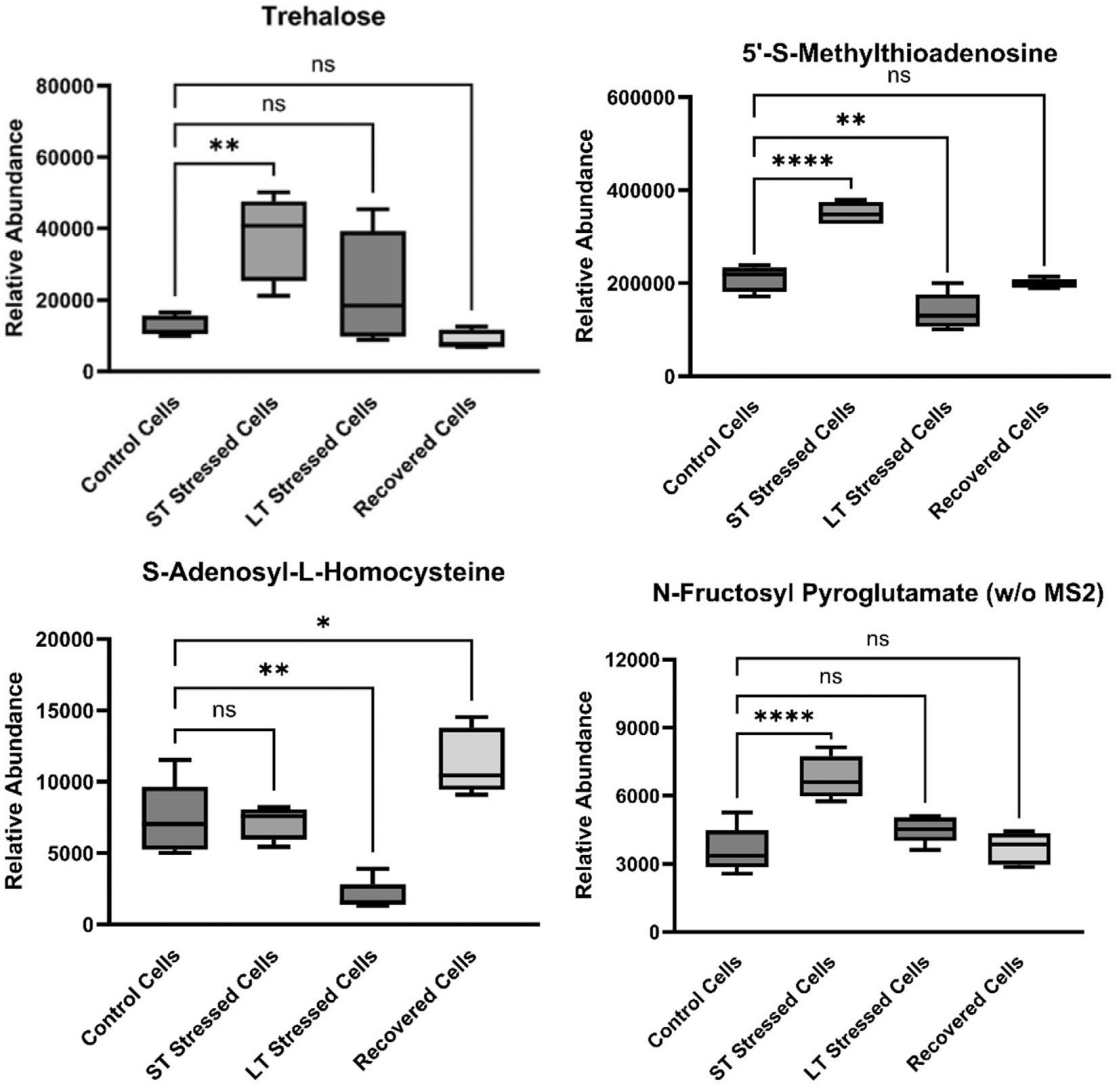
The relative abundance of representative metabolites detected by LC–MS/MS in *Saccharomyces cerevisiae* cells differentially exposed to benzoic acid. The annotation ns indicates the relevant samples were not significantly different. Asterisks indicate the relevant samples were significantly different (*p < 0.05, **p < 0.01, ****p < 0.0001). Data represent mean ± SD of five biological replicates per condition. Statistical significance was assessed using one‐way ANOVA followed by Tukey's post hoc test for multiple comparisons.

The major findings of this study are presented in Figure 1, which summarizes transcriptomic and metabolomic results in terms of the up‐ and downregulation of key genes and metabolites in all treated yeast cells compared to the Control Cells. One clear finding from the metabolomic analysis was increased production of SAH, which is shown in both Figure 2 (metabolite 12 [negative charge mode], and metabolites 40 and 41 [positive charge mode]) and Figure 3, in Recovered Cells compared to Control Cells. Both figures depict the metabolic profiles in all types of yeast cells, as detected by LC–MS/MS analysis, with Figure 2 presented as a circular heatmap and Figure 3 emphasizing four key metabolites. Interestingly, the SAH production was significantly downregulated in LT Stressed Cells (opposite of Recovered Cells). Activation of SAH production is likely necessary for cell survival after long‐term stress exposure. Our findings align with the recent study by Poganik et al. (2023), who utilized DNA methylation‐based epigenetic clocks to quantify biological age in mice and humans exposed to physiological stressors, including surgery, severe infection and pregnancy. Their work demonstrated that biological age temporarily increases during stress and is restored upon recovery, establishing that aging is not strictly unidirectional at the molecular level. In a complementary perspective, Poganik and Gladyshev (2023) argued for shifting the focus of aging research to the underlying molecular process itself, rather than defining aging solely through downstream consequences like mortality. While our study measures chronological lifespan and transcriptomic/metabolomic profiles rather than epigenetic age, the conceptual parallel remains that stress induces molecular changes associated with aging that are at least partially reversible upon stress removal (Poganik et al. 2023). The upregulation of SAH production plays a pivotal role in aiding cell recovery following exposure to stress. Therefore, our findings indicate that SAH levels in a cell could serve as a biomarker for recovery. Among the metabolites detected, N‐fructosyl pyroglutamate showed differential abundance across conditions (Figure 3). This Amadori compound, formed by nonenzymatic glycation of pyroglutamic acid, has been identified as a marker of the Maillard reaction and may reflect altered amino acid and sugar metabolism under stress. Its accumulation in stressed cells is consistent with increased nonenzymatic protein modification, a process linked to proteostatic decline during aging.

**FIGURE 4:**
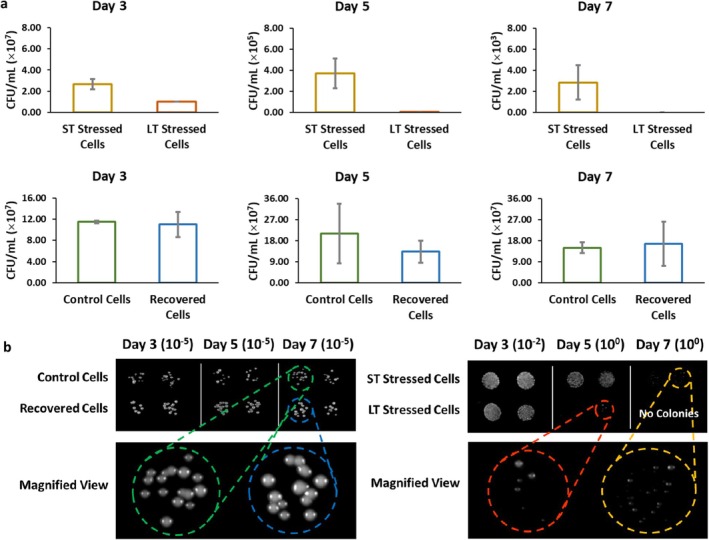
Chronological aging experiment of *Saccharomyces cerevisiae* exposed to different stress durations. (a) Cell viability (*p* < 0.05 between ST Stressed Cells and LT Stressed Cells, *p* > 0.05 between Control Cells and Recovered Cells) and (b) morphology on Day 3, 5, and 7. The initial colony‐forming units (CFU/mL) of each sample were normalized on Day 0, at 10^6^ CFU/mL. Panels in (b) show colony morphology at each time point, with magnified views shown in adjacent panels. Control and recovered cells were compared as one group, while ST and LT stressed cells were compared as a separate pair. For the stressed groups, Day 0 of the CLS assay corresponds to the completion of their respective stress exposures (24 h for ST and 500 h for LT), after which they remained under the same stress condition for up to 7 days during the assay. For morphology observation, 10 μL of serially diluted *S. cerevisiae* cells were plated on YPD agar plates, which were then incubated at 37°C for 24 h. Data represent mean ± SD of three independent biological replicates. Statistical significance was assessed by one‐way ANOVA with Tukey's post hoc test.

Yeast cell viability and morphology were evaluated using the chronological lifespan experiment (Figure 4). This showed that LT Stressed Cells have a relatively shorter lifespan than ST Stressed Cells, but that they recovered immediately once the stress was removed from the environment. The morphology of ST and LT Stressed Cells was similar; however, the Recovered Cells exhibited an improved morphological appearance compared to the Control Cells (Figure 4c). The observation that the overproduction of SAH did not significantly affect lifespan contradicts the existing evidence that SAH supplementation/overproduction alone can extend lifespan (Ogawa et al. 2022). Nonetheless, in our work, SAH does appear to play a role in facilitating recovery from long‐term stress exposure and may contribute to slight improvements in cell morphology and a modest increase in chronological lifespan among recovered cells following the removal of stress.

Interestingly short‐term stress did not exert a significant impact on SAH production despite increasing the prevalence of most other metabolites measured (Figures 2 and 3). Included in the metabolites which were over produced in the presence for short‐term stress were trehalose and 5′‐S‐methylthioadenosine (MTA). Trehalose, a well‐established metabolite, is recognized for its ability to enhance lifespan (Honda et al. 2010; Seo et al. 2018; Kyryakov et al. 2012). MTA is a nucleoside derived from S‐adenosyl methionine (SAM) (North et al. 2017; Hevia et al. 2004), known for its beneficial properties, including the suppression of tumors (Li et al. 2019). It has been previously demonstrated that enhancing SAM synthesis can lead to an extended lifespan. SAM is generated from methionine through the action of methionine‐adenosyltransferases (MATs) and functions as a methyl group donor for various biological processes. In the course of this metabolic pathway, SAH is formed in a reversible reaction. SAH has been shown to act as an inhibitor of methyltransferases (Giulidori et al. 1984; Gao et al. 2018; Ogawa et al. 2022). Significantly, both SAH and MTA were downregulated in LT Stressed Cells. These findings lend support to our hypothesis that short‐term stress has the potential to activate mechanisms associated with extending lifespan, aligning with existing literature (Epel and Lithgow 2014; Ristow and Schmeisser 2011). Conversely, the downregulation of SAH production under long‐term stress is consistent with altered one‐carbon metabolism, a metabolic shift that correlates with, but does not alone establish, the activation of aging‐associated processes.

### 2.2 Long‐Term Stress Activates Conserved Stress Regulators and Accelerates Aging

**FIGURE 5:**
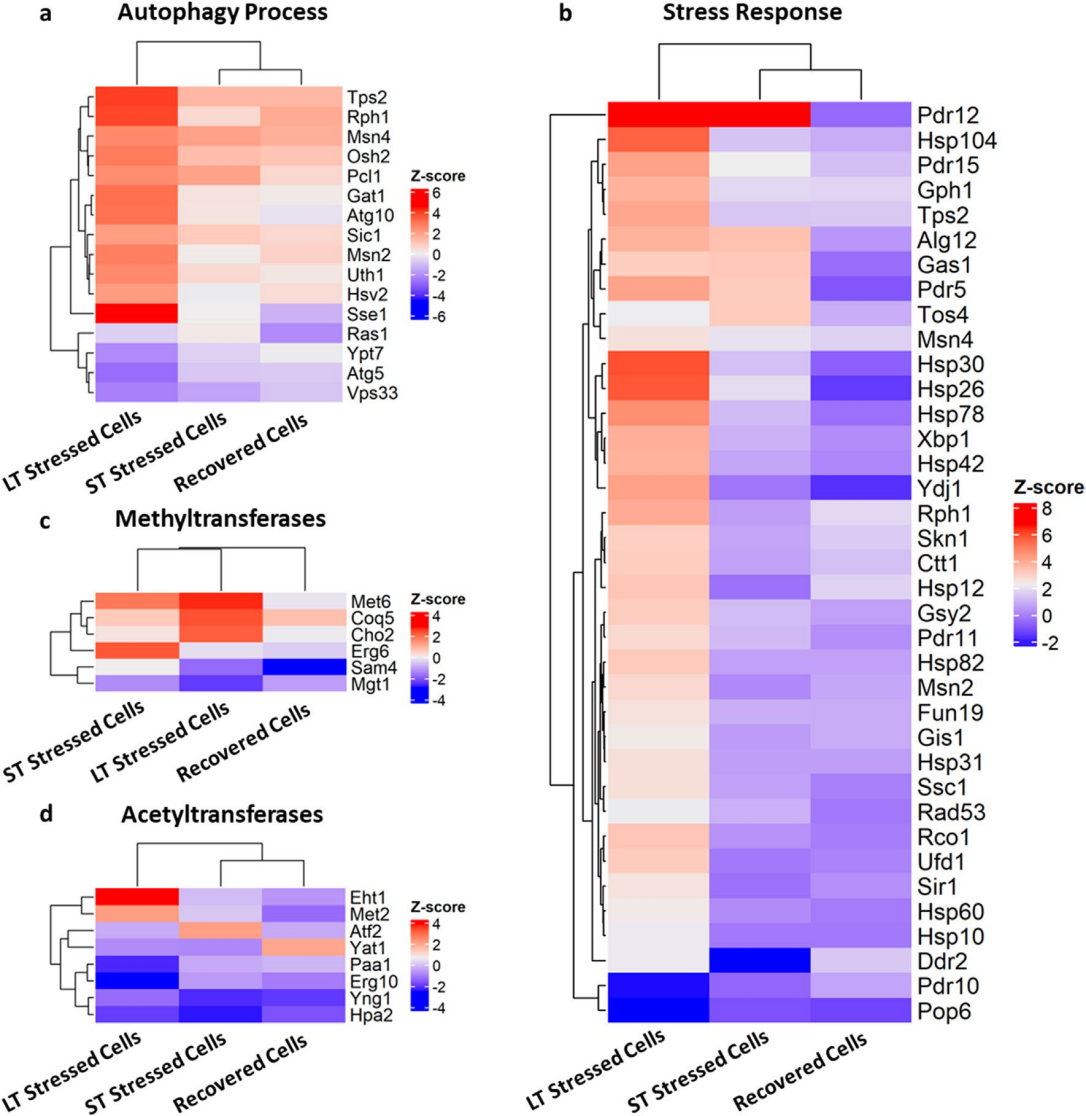
Differentially expressed genes in treated *Saccharomyces cerevisiae* cells in comparison with control cells, which play an important role in: (a) autophagy process; (b) stress response; or in encoding transferases crucial for (c) methylation; and (d) acetylation. *Z*‐score normalization was applied during data visualization, where red and blue color were used to represent up‐ and downregulation of relevant genes.

**FIGURE 6:**
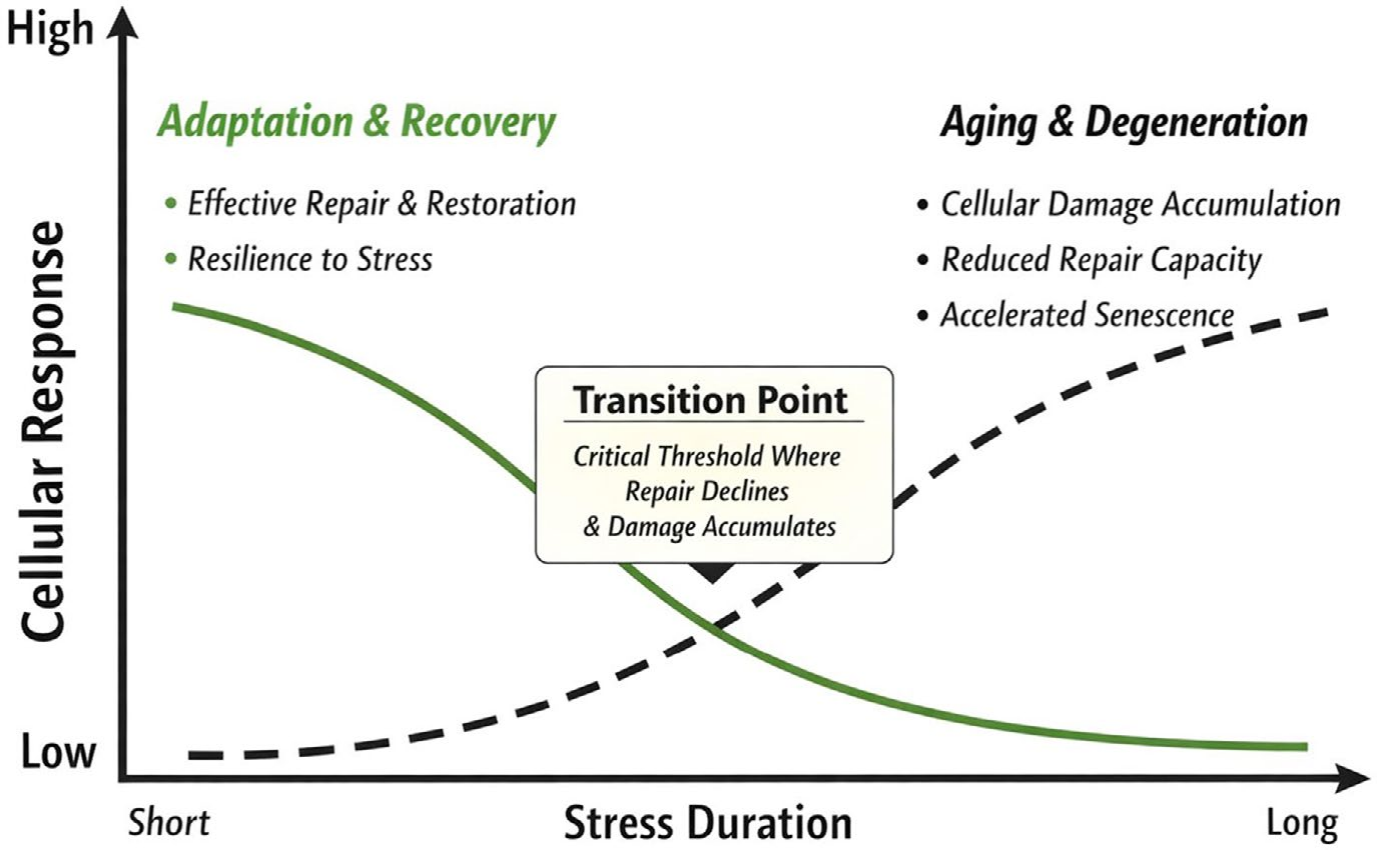
Conceptual model: prolonged stress leads to declining cellular repair and increased aging. This schematic depicts how cellular response (*y*‐axis) changes with increasing stress duration (*x*‐axis). Short‐term stress promotes adaptation and recovery, characterized by robust repair capacity and restored proteostasis. As stress persists, cells reach a transition point, where repair becomes insufficient and damage begins to accumulate. Beyond this threshold, prolonged stress drives aging‐associated phenotypes, including reduced repair efficiency, protein damage accumulation, and accelerated senescence. The model proposes that aging‐related cellular changes emerge when stress adaptation mechanisms are chronically engaged beyond their compensatory capacity.

The gene expression profiles of treated yeast cells, compared to control cells, are presented in Figure 5. This figure includes four categories of genes based on their roles and functions, which are stress response, autophagy, methyltransferases, and acetyltransferases. The cellular defense against stressors such as high temperatures, oxidative stress, or osmotic stress comprises multiple layers of protection. The initial line of defense comprises small molecules with low molecular weight, such as trehalose, activation of essential protein repair systems and chaperones like *pdr12* (Piper et al. 1998) and *pdr5* (Harris et al. 2021) to survive chemical stress, and upregulation of genes like *tos*4 (Cooke et al. 2021) to promote gene expression homeostasis. These components are vital for ensuring immediate survival in adverse conditions. In this study, trehalose, which is a widely known lifespan extension compound (Seo et al. 2018), was significantly overproduced in ST Stressed Cells. This observation supports their dual role in both the first line of defense and also in lifespan extension in the absence of external stresses. The continuation of stress exposure then activates secondary processes such as upregulating the genes responsible for producing protective factors. This transition may mark the onset of aging‐associated changes, accompanied by the activation of secondary stress response effectors such as heat shock proteins (Figure 6).

Heat shock proteins (HSPs) are highly prevalent and evolutionarily conserved protein groups found in both prokaryotes and eukaryotes. They preserve the stability of cellular proteins and mediate cellular responses to stress. Heat shock proteins (HSPs) are evolutionarily conserved molecular chaperones classified primarily by molecular size and function. Major families include small HSPs (sHSPs) and the large ATP‐dependent chaperones HSP40, HSP60, HSP70, HSP90, and HSP110 (Ban et al. 2019; Hu et al. 2022). There is compelling evidence demonstrating age‐related increases in the expression of HSPs. For instance, in the nematode *C. elegans* , several HSPs with human homologs (e.g., *hsp‐16.48, hsp‐43, hsp‐17*, and *sip‐1*) were found to increase in abundance as the nematodes aged (Manière et al. 2014). Additionally, elevated transcript levels of HSPs were associated with reduced heat shock resistance, disrupted proteostasis, and shorter lifespan in these nematodes (Manière et al. 2014). This paradoxical increase in HSP expression alongside reduced stress resistance reflects a failing compensatory response, in which aging cells upregulate chaperones in an attempt to manage the growing burden of misfolded proteins, but cannot fully restore proteostasis. In *Drosophila*, the aging process was observed to lead to an upregulation of *Hspb8* and *HspA* (Yang and Tower 2009; King and Tower 1999). The expression levels of these HSPs were predictive of the lifespan of adult flies under normal aging conditions and when subjected to heat or oxidative stress. The alteration of HSP levels in relation to advanced age remains a subject of debate. The relationship between HSPs and aging remains debated (Gomez 2021). In yeast, overexpression of the protein aggregation–remodeling factor Hsp104p suppresses accelerated aging and impaired segregation of damaged proteins in *sir2* mutants, demonstrating that proteostasis maintenance can attenuate stress‐induced aging‐associated phenotypes (Erjavec et al. 2007).

This points to a general stress‐sensing mechanism capable of detecting and reacting to various stress forms. Initially, a regulatory element responsive to multiple stress conditions was identified as a heat shock factor‐independent heat shock element in the promoters of *ctt1* (Wieser et al. 1991) and *ddr2* (Kobayashi and McEntee 1993). This element was termed the stress response element (STRE) and has demonstrated its ability to mediate transcription induced by diverse types of stress (Kobayashi and McEntee 1993; Marchler et al. 1993; Schüller et al. 1994; Ruis and Schüller 1995; Martinez‐Pastor et al. 1996). In line with its role in promoting general stress resistance, STRE has been identified as the controller of stress‐inducible transcription in genes responsible for protective functions. The roster of genes under the influence of STRE includes *ctt1*, *ddr2*, *hsp12*, *tps2*, *gsy2*, and *gph1* (Ruis and Schüller 1995; Martinez‐Pastor et al. 1996). The mechanisms responsible for detecting and transducing various stress signals via STREs remain poorly characterized.

We show that long‐term stress activates STRE and activates all the genes (*ctt1*, *ddr2*, *hsp12*, *tps2*, *gsy2*, and *gph1*) under the influence of STRE (Figure 5b). GO enrichment analysis confirmed that LT stress‐specific genes were significantly enriched for terms related to response to stress, protein folding, and carbohydrate metabolic process (Figures S6–S9). KEGG pathway analysis further identified enrichment of the MAPK signaling pathway and protein processing in the endoplasmic reticulum among LT‐specific differentially expressed genes (Figures S10–S13). Previous research has shown that *msn2*, *msn4*, and *gis1* (Martinez‐Pastor et al. 1996; Wei et al. 2009; Fabrizio et al. 2001; Fontana et al. 2010) are essential for the activation of numerous yeast genes like *ctt1*, *ddr2*, and *hsp12*, whose upregulation occurs via STREs, and mutants deficient in *msn2* and *msn4* are known to be hypersensitive to severe stress conditions (Moskvina et al. 1998). These genes encode a DNA‐binding component of the stress‐responsive system, and it is probable that they function as positive transcription factors. All three *msn2*, *msn4*, and *gis1* are significantly upregulated in LT Stressed Cells. We also noted a substantial rise in the levels of another HSP gene, *hsp104*. Research has indicated that *hsp104* consistently elevates its concentration as cells age. The concentration of *hsp104* followed a steady increase throughout the aging process, reaching a twofold increase compared to young cells at the time of the final budding event (Moreno et al. 2019). Notably, this increase continued even beyond this point, with *hsp104* concentrations rising further during the subsequent posterior G1 arrest phase at an even higher average rate (Moreno et al. 2019). Several other HSP genes, such as *hsp30*, *hsp26*, *hsp78*, *hsp42*, *hsp12*, and *hsp82* are all overexpressed in LT Stressed Cells (Figure 5b). No significant overexpression of HSP genes was observed in ST Stressed Cells, and downregulation of these HSP genes was observed in Recovered Cells. HSPs have been linked to both cancer and neurodegenerative diseases, and it is well‐established that inhibitors targeting these proteins have demonstrated efficacy against these conditions (Meng et al. 2018; Calderwood and Gong 2016; Zhang, Jing, et al. 2020). Exploring these HSP inhibitors may hold promise as a potential approach for intervening in the aging process.

Furthermore, Labunskyy et al. made an intriguing observation of unexpected lifespan extension in unfolded protein response (UPR) target gene deletions, such as *alg12*. This discovery contradicted initial expectations, as these genes are typically deemed crucial for restoring ER homeostasis and bolstering cellular protection. The authors attributed this surprising outcome to hormesis, where mild stress triggers protective mechanisms against more severe age‐related stressors (Labunskyy et al. 2014). This aligns with our hypothesis that aging represents a long‐term stress response and adaptation, with the deletion of these genes contributing to lifespan extension. In our investigation, we found a significant upregulation of *alg12* in both ST and LT Stressed Cells, with fold increases of 3.4 and 3.8, respectively (see Figure 5b) indicating that increased upregulation can result in a reduced lifespan, while its deletion leads to an extension of lifespan (Labunskyy et al. 2014). The mechanisms underlying lifespan extension due to UPR target gene deletion appear distinct from those associated with increased *sir2* activity or reduced mTOR signaling, which mimic the effects of dietary restriction (Labunskyy et al. 2014).

Our findings also revealed no significant increase in *sir2* activity or *sch9* and only a slight reduction in mTOR activity, indicating the potential existence of separate pathways for nutrient and stress sensing in the context of aging and lifespan extension. In addition, *rad53* has previously been associated with aging but had not been connected to the response to dietary restriction. It is established that *rad53* induces cell cycle arrest in response to DNA damage caused by MMS (Delaney et al. 2013; Sidorova and Breeden 1997; McCormick et al. 2015). Importantly, in our research, we observed a significant upregulation of *rad53* expression in LT Stressed Cells (Figure 5b).

### 2.3 Epigenetic Regulators Link Prolonged Stress to Hallmarks of Aging

Epigenetic mechanisms are integral to the stress response (Stankiewicz et al. 2013). There is substantial evidence endorsing the idea that epigenetic influences play a pivotal role in controlling the aging process. For instance, research has identified the occurrence of epigenetic changes as individuals age, known as “epigenetic drift.” Importantly, the aging rate can be directly influenced by a combination of environmental and epigenetic factors. Chronic exposure to environmental stressors drives epigenetic alterations at both cellular and organismal levels (Zhang, Qu, et al. 2020). Chromatin‐modifying enzymes employ cellular metabolites as their substrates, thus establishing a connection between metabolic pathways and the processes of epigenetic modification and gene regulation. The one‐carbon cycle, mediated by enzymes like MATs, regulates gene expression through epigenetic mechanisms, with SAM and SAH acting as activators and inhibitors, respectively, of DNA and histone methylation. This metabolic control of chromatin dynamics plays a pivotal role in various biological processes (Greco et al. 2020; Li et al. 2018), including aging.

Research has demonstrated that inducing temporary hormetic mitochondrial stress, which includes an elevation in mitochondrial reactive oxygen species (mtROS), triggers advantageous reactions that prolong lifespan. This pathway detects mtROS differently from the response to nuclear DNA damage and, in the end, enhances longevity by deactivating the histone demethylase Rph1, particularly within the subtelomeric heterochromatin region demonstrating a communication pathway linking mitochondria and telomeres, which play a role in governing the aging process and lifespan (Schroeder et al. 2013). In our study, we observed the activation of *rph1* in cells exposed to prolonged stress (toxic stress) over an extended period (as depicted in Figure 5a), indicating it may play a role in extending lifespan during short‐term stress and contributing to aging during long‐term stress exposure.

Research has established that the HDA complex, categorized as a class‐II histone deacetylase (HDAC), governs the aging process by influencing stress response pathways, especially those involved in DNA damage and osmotic stress response. Inhibiting HDA can lead to increased lifespan by inducing the activation of the trehalose metabolic pathway (Figure 5; Yu et al. 2021).

This discovery presents intriguing possibilities for addressing age‐related diseases (Yu et al. 2021), particularly in light of the existing use of multiple HDAC inhibitors for cancer treatment (Yu et al. 2021; Khan and La Thangue 2012). This study observed the activation of the components of the HDA complex in LT Stressed Cells, and this activation is likely to have had an adverse effect on longevity, potentially resulting in accelerated aging in these LT Stressed Cells (Figure 5). One example component of the HDA complex that was activated is Xbp1. Xbp1 functions as a transcriptional repressor that remains dormant during the logarithmic growth phase, yet it becomes activated in response to a wide range of stress stimuli and plays a role in the repair of DNA double‐strand breaks in yeast through Xbp1‐dependent histone H4 deacetylation (Tao et al. 2011). In quiescent cells, *xbp1* transcripts are the most abundant and have the capability to repress around 15% of all yeast genes as the cells transition into the quiescent state. Importantly, numerous studies in higher eukaryotes have revealed the induction or detection of *xbp1* expression in various types of cancer cells (Chen, Chen, et al. 2020), as well as in conditions such as stroke (Lõhelaid et al. 2022) and neurodegenerative diseases, including Alzheimer's disease (Taylor 2016). The overexpression of *xbp1* has demonstrated certain advantageous effects in the aging of the mammalian brain (Cabral‐Miranda et al. 2022; Taylor 2016). In our research, we observed a significant increase in the expression of *xbp1* in LT Stressed Cells. Given its induction across aging‐associated conditions, the upregulation of *xbp1* may serve as a marker of aging‐associated stress adaptation.

The gene responsible for Rco1, which forms a homodimer in the Rpd3s histone deacetylate complex (McDaniel et al. 2016), was also expressed significantly higher in LT Stressed Cells compared to ST Stressed Cells and Recovered Cells (Figure 5b). These findings are consistent with the interpretation that HDAC activation is associated with reduced chronological lifespan under prolonged stress, though a direct causal link requires further validation.

Furthermore, numerous chromatin‐modifying enzymes, including methyltransferase genes like *met6*, *coq5*, *cho2*, and acetyltransferase genes such as *eht1* and *met2*, exhibited significantly higher expression levels in LT Stressed Cells as compared to both ST Stressed Cells and Recovered Cells (Figure 5). Coq5 catalyzes the sole C‐methylation process involved in the formation of coenzyme Q (Q or ubiquinone) in both *S. cerevisiae* and humans. In the yeast Q production pathway, Coq5 is 1 of 11 essential polypeptides and participates in the assembly of the CoQ‐synthome, a multi‐subunit complex. In humans, mutations in *COQ* genes result in primary Q deficiency, and reduced CoQ biosynthesis is associated with mitochondrial, cardiovascular, kidney, and neurodegenerative disorders (Nguyen et al. 2014). Previous investigations in yeast have revealed that specific point mutations located within or near the conserved methyltransferase motifs of *coq5* result in the impairment of Coq5 methyltransferase function. Mutants carrying these specific alleles (*coq5‐2, coq5‐5*) retain consistent levels of Coq5 protein, and supplementation with *ubiE*, the *Escherichia coli coq5* analog, restored respiration and C‐methyltransferase activity (Barkovich et al. 1997; Baba et al. 2004).

The introduction of human *COQ5* into yeast mutants by Nguyen et al. (2014) demonstrates functional conservation between yeast and human Q biosynthesis pathways, with direct relevance to the diagnosis and treatment of Q10 deficiencies in patients.

Methylation‐driven gene silencing is crucial in colorectal and pancreatic cancer progression (Wang et al. 2024). Hsp90 regulates DNA methyltransferase (DNMT) enzymes. Inhibiting Hsp90 with ganetespib reduced DNMT1, DNMT3A, and DNMT3B levels in HT‐29 and MIA PaCa‐2 cells, leading to reduced DNA methylation and gene reactivation (Nagaraju et al. 2017). Expression levels of Hsp60 are heightened in various types of cancer (Meng et al. 2018). Furthermore, translational modifications of Hsp60, such as acetylation, have been observed in several cancer types (Caruso Bavisotto et al. 2020). Additionally, Hsp70 chaperones have been implicated in cancer, inflammation, and Alzheimer's disease (Broer et al. 2011). Epigenetic modifications are known to regulate the Hsp70 pathway (Ban et al. 2019).

In this study, both Hsp60 and *ydj1*, which discriminate among Hsp70 isoforms and facilitate substrate transfer to Hsp70, were significantly expressed in cells subjected to prolonged stress. Targeting HSPs therefore represents a potential strategy for treating age‐related diseases and attenuating the aging process. Recovered Cells displayed a distinct transcriptional profile characterized by downregulation of HSPs and STRE‐regulated genes relative to LT Stressed Cells, alongside restored levels of SAH and other protective metabolites (Figure 1). Gene Ontology enrichment analysis of differentially expressed genes in Recovered Cells revealed reactivation of biosynthetic and metabolic processes, consistent with a return toward the control transcriptional state (Figures S6–S9). This partial transcriptional reversal, coupled with the improved chronological lifespan of Recovered Cells, supports the interpretation that the LT stress‐associated molecular program is at least partially reversible. Stressor removal permits partial restoration of a prestress gene expression and metabolic profiles, aligning with the reversibility of biological age observed by Poganik et al. (2023).

### 2.4 Autophagy Suppression Under Long‐Term Stress Contributes to Aging

Autophagy, a cellular process for recycling, is triggered by stressors like nutrient scarcity, viral infection, and genotoxic stress. Recent evidence points to oxidative stress, mediated by reactive oxygen species (ROS) and reactive nitrogen species (RNS), as the common signal driving autophagy (Filomeni et al. 2015). While several potential mechanisms linking oxidative stress to autophagy have been proposed, few have been confirmed to influence autophagy. Clarifying the molecular control of autophagy by ROS and RNSmay inform the development of anticancer and targeted therapies (Filomeni et al. 2015).

Overexpression of *ATG5* activates autophagy and prolongs the lifespan of mice while enhancing their resistance to oxidative stress. Notably, this increased tolerance to oxidative stress can be reversed by the use of any autophagy inhibitor. The initial genetic link between autophagy and aging was established in *C. elegans* , where the reduction of *beclin1* in *daf‐2* mutants dramatically shortens their lifespan (Pyo et al. 2013). In our study, *atg5* was significantly downregulated in LT Stressed Cells (Figure 5a), suggesting that reduced autophagic flux may contribute to aging‐associated phenotypes in these cells. However, this observation is inconsistent with our hypothesis that the molecular pathways activated during long‐term stress adaptation overlap with conserved stress‐survival functions found across all domains of life. Autophagy is not conserved in prokaryotes. Similarly, telomere attrition and telomerase genes, while relevant to aging, are not conserved in prokaryotes. One explanation is that autophagy and telomere maintenance represent eukaryote‐specific stress response mechanisms. Alternatively, they may represent secondary stress adaptations that have evolved in eukaryotes to support the demands of complex multicellular life.

### 2.5 Evolutionary Conservation of Stress–Aging Regulators

**FIGURE 7:**
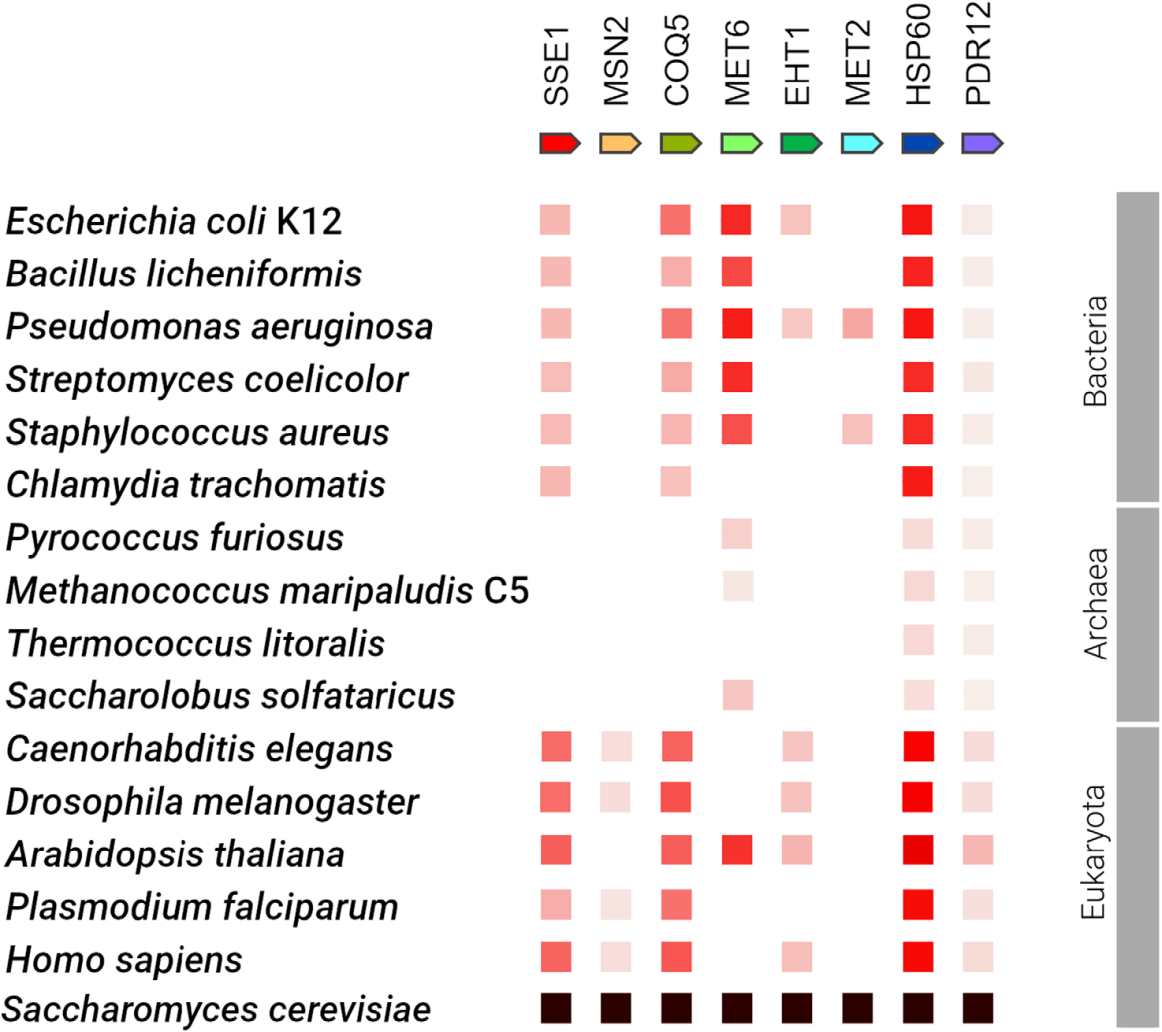
Gene co‐occurrence of all regulated genes across all domains of life. The colored square denotes, for each gene, the similarity of its best hit in a given STRING genome. The intensity of the squares denotes the degree of similarity: the more intense the square is, the more similar the gene is to its best hit. The highest intensity means a 100% sequence conservation. Black indicates the gene used for comparison.

Genes exclusively activated in ST Stressed Cells are conserved solely in eukaryotes, while aging‐related genes significantly expressed in LT Stressed Cells exhibit high conservation across all domains of life, primarily showing close sequence similarity to bacterial homologs. The genome‐wide conservation pattern across differentially expressed genes across all domains of life is shown in Figure 7 and Figure S18. In addition, Figures S1–S5 illustrate the maximum likelihood phylogenetic trees of five highlighted yeast genes: *coq5*, *eht1*, *sse1*, *tor1*, and *hsp60*, along with their homologs across different domains of life. Among these genes, notable candidates include secondary stress response genes such as *hsp60* and the epigenetic modifying enzyme *coq5*. The presence of *hsp60* homologs in various life domains and phylogenetically distant organisms raises questions about their origin, suggesting either a common ancestral source or horizontal gene transfer between different lineages. Additionally, well‐supported clades of Archaea homologs of *hsp60* further emphasize the widespread distribution of this gene. Similarly, the closest human homolog of yeast *hsp60*, the *HSPD1* gene, forms a well‐supported monophyletic clade with the yeast *hsp60* gene (Figure S6). Likewise, *coq5* homologs display a wide distribution across life domains, posing the same questions about their origin and suggesting a potential horizontal gene transfer. Yeast *coq5* homologs are most commonly found in the bacterial phylum Proteobacteria. Moreover, the closest human homolog of yeast *coq5*, the *TNT1A* gene, forms a well‐supported monophyletic clade with homologs from the bacterial phyla Proteobacteria and Firmicutes, hinting at a conserved genetic relationship between the yeast *coq5*, the human *TNT1A*, and analogous genes present in bacteria (Figure S1). This conservation of stress response genes across different organisms and domains of life highlights the deep evolutionary origins of these stress‐survival pathways, although their conservation most likely reflects selection on essential cellular functions rather than on aging itself.

### 2.6 Biotechnological Implications of Stress‐Induced Metabolite Shifts

Recent findings have shown that the fission yeast *Schizosaccharomyces pombe*, when cultivated at threshold levels of caffeine, developed epimutations (phenotypic changes mediated by unstable silencing rather than DNA alterations). These epimutants displayed cross‐resistance to antifungal agents, suggesting that epigenetic processes can promote phenotype plasticity without modifying the genotype (Feil and Fraga 2012; Torres‐Garcia et al. 2020). Yeast alcoholic fermentation can similarly be modified by epigenetic modifiers (Kong et al. 2022). In this study, we have demonstrated that we can alter yeast metabolism using the epigenetic modifier, benzoic acid, resulting in the differential production of 61 metabolites detected as shown in Figure 2. Among these metabolites, several have been revealed to activate the immune response, including nucleosides such as guanosine‐3′,5′‐cyclic monophosphate (metabolite 49, Figure 2; Tal et al. 2021; Verrier and Langevin 2021) and their analogs (Bernheim et al. 2021; Lee et al. 2003) which are known to act as antiviral molecules against several human viruses by acting as a chain terminator for the RNA polymerase (Bernheim et al. 2021). In addition, pyroglutamic acid (Celik et al. 2023), piperine, and trehalose (Nag and Chowdhury 2020; Ai et al. 2022; Wu et al. 2015) are known for their antiviral activity. SAH is also a well‐known antiviral compound whose accumulation is suppressed during viral infection (Bray et al. 2000); its downregulation in adapted cells may reflect an analogous adaptive response. There is the potential then to use this approach to increase production of metabolites with biotechnology applications such as the essential amino acid L‐phenylalanine which was shown to be produced in higher quantities in both ST and LT Stressed Cells (Khamduang et al. 2009; Figure 2).

## 3 Conclusion

ST Stressed Cells activate genes such as *pdr12* and *alg12*, and metabolites such as SAM and trehalose, which provide immediate protection from stress and potential lifespan extension. In contrast, prolonged stress activates secondary stress response genes, including heat shock proteins, whose expression patterns are consistent with aging‐associated phenotypes and may reflect a cell stress adaptation and survival mechanism. Primary stress response genes like *pdr12* are also expressed in LT Stressed Cells, which potentially help in the recovery of cells when stress is removed.

This study provides evidence that long‐term stress is associated with aging‐like phenotypes in *S. cerevisiae*, and we propose that these phenotypes emerge when stress adaptation mechanisms, beneficial for short‐term survival, are chronically engaged beyond their compensatory capacity. The evidence presented in this study further indicates that stress‐associated aging phenotypes observed here are at least partially reversible as demonstrated by the improved lifespan of the Recovered Cells. This is conceptually consistent with Poganik et al. (2023), who demonstrated the reversibility of biological age following the removal of stressors. Furthermore, we provide evidence that primary stress response genes such as *pdr12* and *alg12*, and metabolites such as SAH and trehalose, are activated during short‐term stress, while secondary response genes such as *hsp12* and *hsp60* are activated during long‐term stress. Stress response and longevity‐related genes like *tor1* are conserved across eukaryotes, but absent or poorly conserved in prokaryotes. On the other hand, genes upregulated under prolonged stress, including methyl transferase *coq5* and secondary stress response genes such as *hsp60*, are conserved across all life domains (Figure 7).

One of the most well‐established concepts in biology is that all life undergoes change through natural selection. Our results suggest that the stress response pathways associated with aging‐like phenotypes in LT Stressed Cells are conserved across domains of life, most likely reflecting selection on essential stress‐survival functions rather than having evolved specifically to cause aging. Finally, benzoic acid, a known epigenetic modifier, increased production of several beneficial metabolites under stress conditions, including antiviral compounds with clinical potential, illustrating the utility of epigenetic modification as a non‐GMO approach for directed metabolic engineering.

Whether aging‐associated changes are a consequence of long‐term stress adaptation, or themselves constitute a survival mechanism under prolonged stress, requires additional investigation. Although nutrient‐ and stress‐sensing pathways are interconnected (Sung et al. 2023), this study did not resolve the relationship between them in the context of stress‐induced aging. For instance, *tor1* was not significantly downregulated in recovered cells, suggesting that nutrient and stress sensing may act through a partially independent mechanism. This study focused on a single chemical stressor, which may not capture the full spectrum of aging responses; future work incorporating thermal, oxidative, and dietary stressors will be needed to determine the generality of these findings. Additionally, while ST cells continued to experience stress during the CLS assay, which may influence long‐term outcomes, the consistent direction of the transcriptomic and metabolomic changes across conditions supports the robustness of the overall trends. Although functional validation of individual genes was not performed, the integrative multi‐omics approach adopted here provides a systems‐level perspective that single‐gene studies cannot achieve.

## 4 Materials and Methods

### 4.1 Stress Conditions on Model Strain

A commercial yeast strain, *S. cerevisiae* EC‐1118 (Lalvin EC1118), was used as the model strain in this study. Untreated wild‐type *S. cerevisiae* cells served as Control Cells. Short‐term (ST) Stressed Cells were obtained by exposing *S. cerevisiae* to 10 mM benzoic acid for 24 h. In contrast, Long‐term (LT) Stressed Cells were produced by continuously culturing yeast in 10 mM benzoic acid for 500 h, with subculturing every 24 h to maintain exponential‐phase viability. To generate Recovered Cells, LT Stressed Cells were transitioned out of the stress condition by harvesting the cultures via centrifugation (3000 × g, 3 min), washing the pellets twice in sterile 1× PBS, and resuspending them in fresh YPD broth at a defined starting density (~10^6^ CFU/mL). These resuspended cultures were then grown in YPD for 16 h to allow physiological recovery in the absence of benzoic acid.

### 4.2 RNA Preparation for Transcriptome Sequencing

*S. cerevisiae* RNA samples were purified using the RiboPure RNA Purification Kit for yeast (Thermo Fisher Scientific Inc., Waltham, MA, USA), following the manufacturer's instructions. Subsequently, the RNA samples were air‐dried in a biosafety hood for 20 h and preserved in RNA stabilization tubes (GENEWIZ, South Plainfield, NJ, USA) at room temperature before being shipped to Azenta Life Sciences (Suzhou, China) for transcriptome sequencing and analysis. The sequencing was carried out on the Illumina Novaseq platform, using a 2 × 150 bp paired‐end (PE) configuration, with approximately 9.0 Gb of PF data per sample. In total, approximately 108.0 Gb of PF data was generated for the 12 *S. cerevisiae* RNA samples, with triplicate samples for each treatment.

### 4.3 mRNA Library Construction and Sequencing

A total of 1 μg of RNA was used for library preparation. Poly(A) mRNA isolation was performed using Oligo(dT) beads. mRNA fragmentation was achieved using divalent cations and high temperature. Random primers were used for priming. First‐strand cDNA and second‐strand cDNA were synthesized. The purified double‐stranded cDNA was then treated to repair both ends, and dA‐tailing was added in a single reaction, followed by a T‐A ligation to attach adaptors to both ends. Size selection of adaptor‐ligated DNA was performed using DNA Clean Beads. Each sample was amplified by PCR using P5 and P7 primers, and the PCR products were validated. Libraries with different indexes were multiplexed and loaded onto an Illumina HiSeq, Illumina Novaseq, or MGI2000 instrument for sequencing, using a 2 × 150 paired‐end (PE) configuration following the manufacturer's instructions.

### 4.4 Transcriptomic Data Analysis

#### 4.4.1 Quality Control

To eliminate technical sequences such as adapters, polymerase chain reaction (PCR) primers, or their fragments, and to filter out bases with a quality score lower than 20, the pass filter data in fastq format were processed using Cutadapt (V1.9.1). The parameters used for this processing included a phred cutoff of 20, an error rate of 0.1, an adapter overlap of 1 bp, a minimum length of 75, and a maximum proportion of N of 0.1. This resulted in the generation of filtered, adapter‐free reads suitable for downstream analysis (Martin 2011).

#### 4.4.2 Alignment

First, reference genome sequences and gene model annotation files from relevant species were downloaded from genome websites like UCSC, NCBI, and ENSEMBL. Then, the reference genome sequences were indexed using Hisat2 (v2.0.1). Finally, the clean data were aligned to the reference genome using Hisat2 (v2.0.1). Hisat2 implements a graph‐based alignment algorithm optimized for spliced alignment of RNA‐seq reads (Kim et al. 2019).

#### 4.4.3 Expression Analysis

Initially, transcripts in FASTA format were generated from a known GFF annotation file and correctly indexed. Then, using this file as the reference gene file, HTSeq (v0.6.1) estimated gene‐level expression by counting reads mapping to annotated features using the union mode (Anders et al. 2015). Expression values were reported as raw read counts, which were subsequently normalized by DESeq2's median‐of‐ratios method during differential expression analysis.

#### 4.4.4 Differential Expression Analysis

Differential expression analysis was performed using the DESeq2 Bioconductor package (Love et al. 2014), which models count data using a negative binomial distribution with shrinkage estimation for dispersion and fold change. Genes with a Benjamini–Hochberg adjusted *p* value < 0.05 and an absolute log_2_ fold change > 1 were considered differentially expressed.

#### 4.4.5 GO and KEGG Enrichment Analysis

GOSeq (v1.34.1) was used to identify Gene Ontology (GO) terms annotating a list of enriched genes with a significant *p*‐adj < 0.05. Additionally, we utilized topGO to create Directed Acyclic Graphs (DAGs). KEGG (Kyoto Encyclopedia of Genes and Genomes) is a collection of databases encompassing genomes, biological pathways, diseases, drugs, and chemical substances (Kanehisa et al. 2023). We employed in‐house scripts to enrich significantly differentially expressed genes in KEGG pathways.

### 4.5 Cell Lysates Preparation for LC–MS/MS

*S. cerevisiae* cells were freshly grown in YPD broth overnight. Cell quenching was achieved by transferring 1 mL of cultured broth to a tube containing 4 mL of precooled MeOH/ddH_2_O (60:40) with 10 mM ammonium acetate. The mixture was promptly placed at −80°C for 2 min, followed by centrifugation at 4000 rpm for 5 min at −10°C. Intracellular metabolites were extracted by resuspending the cell pellets in 1 mL of 80% EtOH, followed by heating to 80°C for a total of 4 min with 10 s of vigorous vortexing in between. Finally, the mixture was centrifuged at 10,000 rpm for 5 min at −10°C to obtain the supernatant containing intracellular metabolites. The extracts were aliquoted and stored at −80°C until analysis using Liquid Chromatography with tandem mass spectrometry (LC–MS/MS), which was conducted by the Proteins & Metabolites Team at AgResearch (Lincoln, New Zealand). The analysis was performed on five biological replicates for each *S. cerevisiae* treatment.

### 4.6 Metabolomic Analysis

#### 4.6.1 Sample Preparation

Samples obtained from the cell lysis protocol were stored at −80°C until analysis. To prepare the samples, they were thawed overnight at 4°C, and 800 μL of ice‐cold methanol:water (1:1) was added to 200 μL of each sample. The samples were then vigorously shaken using a TissueLyzer (Qiagen, USA) and centrifuged for 20 min at 4°C (14,001 × g). Two aliquots, each containing 200 μL of the supernatant, were extracted—one for sample analysis and the other for pooled quality control (QC). The samples, along with 200 μL of the pooled QC samples, were dried down using a vacuum concentrator (Vacuumbrand, Wertheim, Germany) and then reconstituted in acetonitrile:water (1:1). D2‐tyrosine was added as an internal standard to monitor sample degradation.

#### 4.6.2 Chromatography

Samples were analyzed using a Nexera X2 ultra high‐performance liquid chromatography (UHPLC) system (Shimadzu, Japan), which included a SIL‐30AC autosampler coupled to an LCMS‐9030 quadrupole time‐of‐flight (Q‐TOF) mass spectrometer (Shimadzu, Japan) equipped with an electrospray ionization source (see Figure S11). A 2 μL sample was injected into a normal phase Ascentis Express HILIC UHPLC column (2.1 × 100 mm, 2 μm particle size; Sigma, USA) and eluted at 30°C over a 20‐min gradient with a flow rate of 400 μL/min. The mobile phase consisted of solvent A (10 mM ammonium formate in water) and solvent B (acetonitrile with 0.1% formic acid). The solvent gradient program started at 97% solvent B from 0 to 0.5 min, decreased to 70% within 11.5 min, further decreased to 10% from 11.5 to 13.5 min, held at 10% for 1.5 min, increased to 97% B within 1 min, and remained at that concentration until the end of the elution run.

#### 4.6.3 Mass Spectrometry

Full scans (m/z 55–1100) and MS/MS scans for windows spanning m/z 20 were set up for analyses in both positive and negative ionization modes. A total of 42 events were configured with a loop time of 0.85 s. The spray voltage was set at 4.0 kV for positive ionization mode and −3.0 kV for negative ionization mode. The collision energy (CE) was maintained at 23 ± 15 V. The ion source was operated under optimal conditions, including a nebulizing gas flow of 3.0 L/min, a heating gas flow of 10.0 L/min, an interface temperature of 300°C, a drying gas flow of 10.0 L/min, a desolvation line temperature of 250°C, and a heat block temperature of 400°C.

#### 4.6.4 Batch Sequence

All samples were analyzed in a single batch, beginning with positive ionization mode. The sequence started with five blanks, followed by an external standard (Amino acid standard, A9906; Sigma, USA) to verify system performance. Next, a few QC pooled samples were run, and then the actual samples in a randomized order with QC samples interspersed once every eight samples. The same order was followed for negative ionization mode.

#### 4.6.5 Data Analysis

Raw data files (.lcd) were converted to the mzML file format using LabSolutions software (Version 5.99 SP2; Shimadzu, Japan). MS‐Dial was employed for peak detection, MS2 deconvolution, sample alignment, and compound identification (Tsugawa et al. 2015). Appropriate adducts were selected for both positive and negative ionization modes. Blank data was subtracted from all samples, ensuring that the sample's maximum ratio to the average of the blanks was maintained at 5. Local Weighted Scatterplot Smoothing (LOWESS) was used for signal correction during data preprocessing. After preprocessing, only features with metabolite IDs that matched with MS/MS or m/z (without MS/MS) were exported, along with their respective normalized peak areas for statistical analysis. Statistical analysis of the normalized data was performed using MetaboAnalyst 5.0, a web‐based platform for metabolomics data analysis (Pang et al. 2021).

### 4.7 Yeast Viability, Morphology, and Chronological Lifespan (CLS) Assays

Overnight cultures of *S. cerevisiae* were grown in YPD broth and inoculated into baffled flasks at a 5:1 flask‐to‐medium ratio, followed by incubation at 32°C with shaking at 120 rpm. Yeast growth and viability during routine culturing and stress treatments were assessed by measuring optical density at 600 nm (OD_600_) using a FLUOstar Omega microplate reader (BMG LABTECH, Germany) and by determining colony‐forming units (CFUs). For CFU analysis, cultures were serially diluted in sterile 1× PBS (100 μL into 900 μL; 10^0^–10^−6^ dilution series), and 10 μL of each dilution was spotted onto fresh YPD plates. Plates were incubated for 24 h at 32°C, after which colonies were counted to calculate viability (CFU/mL). Colony morphology at defined time points was recorded using a Gel Doc XR+ Imaging System (Bio‐Rad, USA). All viability measurements and morphology assessments were performed using three independent biological replicates, each consisting of technical triplicates.

Chronological lifespan (CLS) of *S. cerevisiae* was assessed using a standard outgrowth‐based assay. For ST Stressed Cells, cultures were exposed to 10 mM benzoic acid for 24 h, whereas LT Stressed Cells were maintained in 10 mM benzoic acid for 500 h, with subculturing every 24 h to preserve exponential‐phase viability during the stress induction period. After completing their respective stress exposures, both ST and LT Stressed Cells remained in benzoic acid stress conditions for the entire CLS assay, ensuring consistency in stress exposure during chronological aging. In contrast, Control and Recovered Cells were maintained in standard YPD without benzoic acid throughout CLS.

At the completion of each stress exposure, cultures were harvested by centrifugation (3000 × g, 3 min), cell pellets were washed twice and resuspended in sterile 1× PBS, and normalized to approximately 10^6^ CFU/mL based on OD_600_ calibration. This normalization step defined Day 0 of the CLS assay for all groups. To minimize contributions from newly budded daughter cells, CLS assays were initiated only after cultures had completed their designated stress exposure and reached a non‐proliferative state. Under the conditions used, baseline doubling times in YPD averaged 1.5–2.5 h, but growth was substantially slower under benzoic acid treatment. No additional subculturing occurred after entry into CLS.

Samples were collected on Days 0, 3, 5, and 7 of CLS. At each time point, serial dilutions were prepared in 1× PBS and plated for CFU determination as described above. In parallel, outgrowth kinetics were monitored by measuring OD_600_ every 30 min using the FLUOstar Omega reader. Viability at each time point was quantified from the delay in outgrowth relative to Day 0 cultures, providing a measure of the proportion of cells capable of resuming growth. All CLS data represent the mean of three independent biological replicates, each averaged from technical triplicates.

### 4.8 Gene Phylogeny

Five genes were selected for full maximum likelihood phylogenetic analysis based on three criteria applied to the list of differentially expressed genes: (i) functional diversity, with one gene representing each major functional category identified in the transcriptomic data (autophagy process: *sse1*; stress response: *tor1* and *skn1*; acetyltransferases: *eht1*; and methyltransferases: *coq5*); (ii) condition specificity, selecting the gene with the highest log_2_ fold change and lowest adjusted *p* value within each functional category under LT stress; (iii) sequence conservation, prioritizing genes encoding proteins with well‐characterized conserved domains suitable for alignment across phylogenetically distant taxa.

The protein sequences of selected upregulated genes (*sse1*, NCBI accession NP_015219.1; *tor1*, NCBI accession NP_012600.1; *hsp60*, NCBI accession NP_014888.1; *eht1*, NCBI accession NP_009736.3; *coq5*, NCBI accession NP_013597.1) were searched against the Swiss‐Prot database using PROSITE with an *E*‐value threshold of 10^−5^ (Kilinc et al. 2023). Additional homologs were identified using the curated databases on SHOOT.bio (Emms and Kelly 2022) and the “top IMG homologs” function on the IMG website (Chen, Chu, et al. 2020). Further homologs were obtained by searching the protein sequences of individual genes against the NCBI nonredundant protein database (nr50) using HMMER (v. 3.3) (Eddy 2011) within the MPI bioinformatics toolkit (Zimmermann et al. 2018), with an *e*‐value threshold of 10^−5^ and retaining the default values of other parameters.

The sequences of homologs obtained for each gene were clustered with DIAMOND‐DeepClust (v. v2.1.3.157) (Buchfink et al. 2021, 2023) within the MPI bioinformatics toolkit, with default parameters. Whenever possible, gene sequences from the Asgard group were used as outgroups, as suggested elsewhere (Zaremba‐Niedzwiedzka et al. 2017). The query sequences of individual genes, clustered homologous sequences, and outgroup sequences were then aligned using MAFFT (v. 7.273) (Katoh et al. 2002), with a gap opening penalty of 3 (Chelikani et al. 2014). The resulting alignment was used as input for phylogenetic tree construction with IQ‐TREE (v. 1.6.12) (Nguyen et al. 2015). The best phylogenetic model was determined using ModelFinder (Kalyaanamoorthy et al. 2017) within IQ‐TREE. The reliability of the nodes in the tree was estimated with 1000 ultrafast bootstraps (Minh et al. 2013) and a maximum of 3000 iterations. Finally, the phylogenetic tree was visualized and annotated using iTOL (v.6.8) (Letunic and Bork 2021).

### 4.9 Gene Co‐Occurrence Pattern

The protein sequences of genes related to the autophagy process, stress response, lifespan/aging, acetyltransferases, and methyltransferases in *S. cerevisiae* S288C were downloaded from the NCBI GenBank database. These gene protein sequences were then input into STRING (Szklarczyk et al. 2015) (https://string‐db.org/) using default parameters to visualize their co‐occurrence across different domains of life.

### 4.10 Data Visualization and Statistical Analysis

Microsoft Office (Microsoft Corporation, Redmond, WA, USA) was used for creating basic graphs. RStudio (Posit, Boston, MA, USA) was employed for generating transcriptomic heatmaps with DESeq2, ggplot2, and ComplexHeatmap packages, as well as multi‐omics graphs using the mixOmics package. Circos version 0.69‐9 was utilized for visualizing metabolomics data. PCA and AHC were analyzed with XLSTAT Statistical Software 2016 (Addinsoft, Paris, France). The statistical analysis was performed with a 95% confidence level, and the data were presented as mean ± SD.

## Author Contributions

Yanzhuo Kong, Damola Adejoro, and Arvind Subbaraj performed the research. Yanzhuo Kong, Christopher Winefield, Stephen L. W. On, Arvind Subbaraj, Andrew Saunders, and Venkata Chelikani analyzed and interpreted the data. Venkata Chelikani conceived the study, design, and overall project management. Yanzhuo Kong, Philip A. Wescombe, and Venkata Chelikani wrote the paper.

## Funding

This work was supported by Callaghan Innovation (70570) and KiwiNet (46561).

## Conflicts of Interest

The authors declare no conflicts of interest.

## Supporting information


**Figure S1:** Maximum likelihood phylogenetic tree of the *Saccharomyces cerevisiae* *coq5* gene and its homologs across different domains of life. The phylogenetic model employed was LG + I + G4, while numbers are ultrafast bootstrap values for selected branches. The branches are colored according to the main phyla/groups. The outer ring is colored according to the main phyla/groups, while branches with dissimilar members are uncolored.
**Figure S2:** Maximum likelihood phylogenetic tree of the *Saccharomyces cerevisiae eht1* gene and its homologs across different domains of life. The phylogenetic model employed was LG + G4, while numbers are ultrafast bootstrap values for selected branches. The branches are colored according to the main phyla/groups. The outer ring is colored according to the main phyla/groups, while branches with dissimilar members are uncolored.
**Figure S3:** Maximum likelihood phylogenetic tree of the *Saccharomyces cerevisiae* *sse1* gene and its homologs across different domains of life. The phylogenetic model employed was LG + I + G4, while numbers are ultrafast bootstrap values for selected branches. The branches are colored according to the main phyla/groups. The outer ring is colored according to the main phyla/groups, while branches with dissimilar members are uncolored.
**Figure S4:** Maximum likelihood phylogenetic tree of the *Saccharomyces cerevisiae* *tor1* gene and its homologs across different domains of life. The phylogenetic model employed was LG + F + I + G4, while numbers are ultrafast bootstrap values for selected branches. The branches are colored according to the main phyla/groups. The outer ring is colored according to the main phyla/groups, while branches with dissimilar members are uncolored.
**Figure S5:** Maximum likelihood phylogenetic tree of the *Saccharomyces cerevisiae* *hsp60* gene and its homologs across different domains of life. The phylogenetic model employed was LG + F + I + G4, while numbers are ultrafast bootstrap values for selected branches. The branches are colored according to the main phyla/groups. The outer ring is colored according to the main phyla/groups, while branches with dissimilar members are uncolored.
**Figure S6:** (a) Principal component analysis (PCA) biplot illustrating the relationship between *Saccharomyces cerevisiae* cell types and known metabolites detected by LC–MS/MS analysis under positive charge mode. (b) *S. cerevisiae* cell types grouped using a agglomerative hierarchical clustering (HCA) according to their dissimilarity levels based on metabolites detected under positive charge mode. (c) Principal component analysis (PCA) biplot illustrating the relationship between *S. cerevisiae* cell types and known metabolites detected by LC–MS/MS analysis under negative charge mode. (d) *S. cerevisiae* cell types grouped using a agglomerative hierarchical clustering (HCA) according to their dissimilarity levels based on metabolites detected under negative charge mode.
**Figure S7:** Circos plot from multiblock sPLS‐DA performed on the *Saccharomyces cerevisiae* RNA sequencing and LC–MS/MS metabolomics data (by Mixomics package in R). The plot represents the correlations greater than 0.9 between variables of different types, represented on the side quadrants. The internal connecting lines show the positive and negative correlations. The outer lines show the expression levels of each variable (gene and metabolite) in each yeast cell type.
**Figure S8:** Diagnostic plot from multiblock sPLS‐DA analysis grouping treated and untreated *Saccharomyces cerevisiae* in terms of the selected genes and metabolites (ncomp. = 3).
**Figure S9:** acel70513‐sup‐0001‐FiguresS1‐S18.docx. *Saccharomyces cerevisiae* samples plot by multiblock sPLS‐DA analysis, the samples are plotted according to their scores on the first two components for each data set. The plot shows the degree of agreement between the different data sets and the discriminative ability of each data set.
**Figure S10:** Comparison of gene expression levels (logFPKM) under different experimental conditions. Each of the five elements in each box plot, from top to bottom, specifies the maximum, upper quartile, median, lower quartile, and the minimum value, respectively. 1‐1, 1‐2, and 1‐3: Control Cells, wild‐type *Saccharomyces cerevisiae* ; 2‐1, 2‐2, and 2‐3: ST stressed cells, short‐term stressed cells, *S. cerevisiae* firstly exposed to 10 mM benzoic acid for 16 h; 3‐1, 3‐2, and 3‐3: LT stressed cells, long‐term stressed cells, *S. cerevisiae* exposed to 10 mM benzoic acid for 500 h (24 h/subculture); 4‐1, 4‐2, and 4‐3: Recovered cells, *S. cerevisiae* exposed to 10 mM benzoic acid for 500 h (24 h/subculture), followed by growing in regular YPD broth for 16 h.
**Figure S11:** (a) Bar graph showing the number of genes significantly up‐ or downregulated in treated groups compared to the control. (b) Venn diagram illustrating the relationships among all treated groups compared to the control.
**Figure S12:** Histogram depicting GO enrichment analysis of short‐term (ST) stressed cells in comparison to control cells. The *X*‐axis represents the number of differentially expressed genes within each GO category. Different categories, such as biological processes, cellular components, and molecular functions, are distinguished by color codes on the right side.
**Figure S13:** Histogram depicting GO enrichment analysis of long‐term (LT) stressed cells in comparison to control cells. The *X*‐axis represents the number of differentially expressed genes within each GO category. Different categories, such as biological processes, cellular components, and molecular functions, are distinguished by color codes on the right side.
**Figure S14:** Histogram depicting GO enrichment analysis of recovered cells in comparison to control cells. The *X*‐axis represents the number of differentially expressed genes within each GO category. Different categories, such as biological processes, cellular components, and molecular functions, are distinguished by color codes on the right side.
**Figure S15:** Scatter plot depicting KEGG enrichment of differentially expressed genes in short‐term (ST) stressed cells compared to the control cells. Dot size corresponds to the number of differential genes within each pathway, showing a positive correlation. Different *Q*‐value ranges are represented by distinct colors.
**Figure S16:** Scatter plot depicting KEGG enrichment of differentially expressed genes in long‐term (LT) stressed cells compared to the control cells. Dot size corresponds to the number of differential genes within each pathway, showing a positive correlation. Different *Q*‐value ranges are represented by distinct colors.
**Figure S17:** Scatter plot depicting KEGG enrichment of differentially expressed genes in recovered cells compared to the control cells. Dot size corresponds to the number of differential genes within each pathway, showing a positive correlation. Different *Q*‐value ranges are represented by distinct colors.
**Figure S18:** Gene co‐occurrence of regulated genes across all domains of life. The heatmap shows the presence and sequence similarity of selected regulated genes across representative species from Bacteria, Archaea, and Eukaryota. The analysis includes eight genes (two genes from each of four functional categories) examined across 16 organisms, including *Saccharomyces cerevisiae* and 15 additional species. For each gene, the colored square represents the similarity of its best hit in a given STRING genome. Color intensity reflects the degree of sequence similarity, with darker shades indicating higher similarity and the highest intensity corresponding to 100% sequence conservation. Black squares denote the reference genes used for comparison. Species are grouped by domain of life, as indicated on the right side of the figure.

## Data Availability

The data that support the findings of this study are available on request from the corresponding author. The data are not publicly available due to privacy or ethical restrictions.
